# Holds enable one-shot reciprocal exchange

**DOI:** 10.1098/rspb.2022.0723

**Published:** 2022-08-10

**Authors:** Marcus Frean, Stephen Marsland

**Affiliations:** ^1^ School of Engineering and Computer Science, Victoria University of Wellington, Wellington, New Zealand; ^2^ School of Mathematics and Statistics, Victoria University of Wellington, Wellington, New Zealand

**Keywords:** reciprocity, cooperation, social dilemma

## Abstract

Strangers routinely cooperate and exchange goods without any knowledge of one another in one-off encounters without recourse to a third party, an interaction that is fundamental to most human societies. However, this act of reciprocal exchange entails the risk of the other agent defecting with both goods. We examine the choreography for safe exchange between strangers, and identify the minimum requirement, which is a shared hold, either of an object, or the other party; we show that competing agents will settle on exchange as a local optimum in the space of payoffs. Truly safe exchanges are rarely seen in practice, even though unsafe exchange could mean that risk-averse agents might avoid such interactions. We show that an ‘implicit’ hold, whereby an actor believes that they could establish a hold if the other agent looked to be defecting, is sufficient to enable the simple swaps that are the hallmark of human interactions and presumably provide an acceptable trade-off between risk and convenience. We explicitly consider the particular case of purchasing, where money is one of the goods.

## 1. Introduction

The direct exchange of objects (where two agents each start with one object and leave with a different one) underlies many quintessentially human behaviours—most obviously, the purchase of goods, but also barter, contract exchange, and the establishment of relationships between groups [1–4]. Any two people with objects can exchange (swap) them should they mutually consent to do so, whether they know one another or not, in one-off encounters [3,5]. However, the ways in which humans perform temporally synchronous exchange (relinquishing control of one good, and gaining it over another), are surprisingly susceptible to abuse, allowing one trading partner to hold both objects at once and potentially defect. Despite this, we perform myriad exchanges daily without thought or concern, very rarely considering the risk involved [6–8]. In this paper, we study the complexity of this fundamental human behaviour, and the extent to which our interactions balance convenience with risk aversion, a question that does not appear to have been considered in the literature.

**Figure 1.**
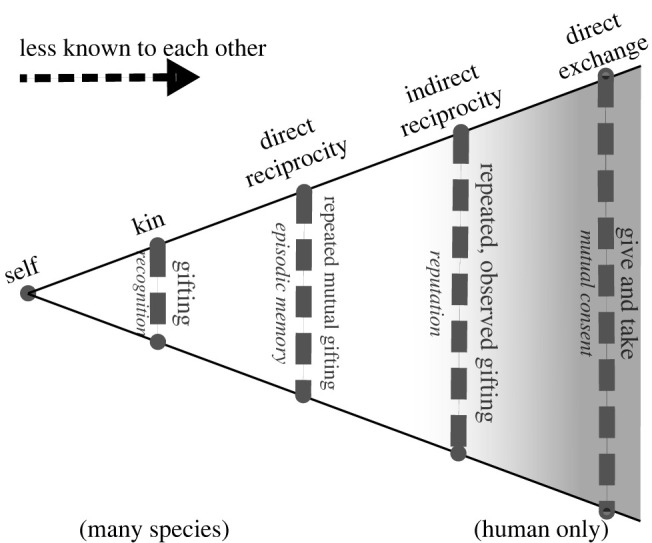
Exchange can be seen as an extension of the evolutionary game theory concept of reciprocity, but is facilitated by a different affordance.

The fact that many exchanges are between strangers, and occur without oversight, is particularly interesting. As is summarized in figure 1, in evolutionary game theory researchers differentiate between kin interactions, direct reciprocity (where agents know one another and interact repeatedly), and indirect reciprocity (repeated interactions between agents that do not know each other directly) [9–11]. Exchanges between kin, and between known agents, are not particularly interesting from the exchange perspective: help between kin is already motivated by inclusive fitness [12], while if agents know each other then defection—cheating to end up with both objects—can be readily penalized. Indirect reciprocity comes closer as an explanation, but is generally solved by providing access to information about how trustworthy the other agent is deemed to be, i.e. a reputation maintained by a trusted third party [13], which is probably one of the reasons why only humans appear to perform this behaviour [11]. While humans can, and do, use a variety of cues to form stereotypical opinions about one another on first sight, these are often inaccurate [14], and exchanges happen regardless (e.g. people frequent street markets where traders they know nothing about proffer goods). We therefore see direct exchange as different to reciprocity: it is one-shot, and does not require any knowledge of the other party.

Our aim here is to study the mechanisms for direct exchange, ask what is required to allow it to occur, and ultimately why the actions that facilitate *safe* exchange (i.e. those that explicitly prevent defection) are not commonly used. We formulate swapping as a competitive game that is played by independent agents. Rather than directly asking the agents to achieve a shared goal of achieving a swap of objects, which has trust built-in, we instead assume that each would prefer to depart with both goods in their possession, since prima facie this is the optimal outcome for either party. We demonstrate that such selfish aims between competing independent agents lead to choreographed swap dances. These dances can be made safe, and with relaxations of the concept of safety, exchanges that resemble typical human behaviours are achieved.

## 2. Model

**Figure 2.**
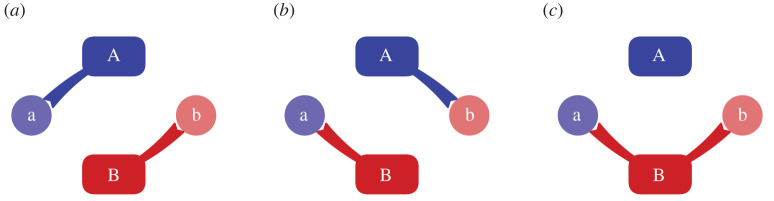
Exchange is not trivial. Suppose two agents hold an object each, but would rather have what the other has. In order to carry out exchange (to ‘swap’ objects a and b; labels highlight which agent has the object originally), agents A and B must negotiate a path from a situation in which each holds an item that the other wants (*a*), towards the state in which they have each acquired the other’s item (*b*). The catch is that they must do this while avoiding states in which one agent would be able to take both items (*c*). (Online version in colour.)

We model independent agents interacting in a form of stochastic game [15] (a competitive Markov decision process [16], updated through value iteration [17]). Pairs of independent agents start with a good that they value less than that held by the other (figure 2*a*) and then interact in an attempt to maximize their payoff. The reward structure has the form familiar from the ‘prisoner’s dilemma’ [18]: mutual cooperation (figure 2*b*) has the highest total payoff, but one of the agents would be better off if they managed to obtain both objects, leaving the other with nothing (figure 2*c*). The decision not to trade leaves each with their original, lower-valued, good. Although the model is non-cooperative, as a general-sum discounted stochastic game it admits a Nash equilibrium (i.e. a point where neither player gains from a change in strategy) [16] at the mutual compromise where there is a safe exchange.

Our game is formulated so that two agents each begin in possession of a good that they perceive to have lower value to them than that held by the other (we investigate variants of this value system in the computational model). Each agent has the potential for three ‘holds’. By holds, we mean some way to prevent the other agent using the object (e.g. by maintaining a physical grasp of it). For objects that are small, such as money—a mechanism that has been developed precisely to facilitate exchange—shared holding is not possible, so we consider the alternative of holding the other agent (i.e. physically stopping them from leaving). Since an agent can potentially have a hold (or not) on three objects, they each have eight states, and so the joint state space has 2^6^ = 64 states. Not all states are achievable, depending upon the parameters chosen. The state *s* at each iteration can be described using 6 binary digits. The first 3 bits encode what agent A is holding (object a, agent B, object b), and the second set do the same for agent B (object a, agent A, object b). Thus, the start state is 100 : 001 and the desired final state is 001 : 100 (cf. figure 2*a*,*b* respectively). The space is small, and can be completely explored.

There are five possible actions d∈A: an agent can ‘toggle’ its hold on either object, or the other agent (toggle means to take a hold on that object if it is not currently held, or let it go), or can opt to pass, or attempt to leave. If the agent attempts an action that is forbidden, the state is unchanged. Regardless of which agent performs an action, both agents potentially receive rewards and incur costs at every step.

The only positive rewards are the values of the two objects. These are controllable parameters. By default, they were set to 4 for the other agent’s good (b for agent A and a for agent B) and 1 for their own, chosen to match the ‘classic’ linear Prisoners Dilemma payoffs. Thus, the maximum payoff would be five, for leaving with both goods. We also considered costs (negative rewards), which might be incurred by (i) establishing a hold on an object or agent, (ii) maintaining a hold, (iii) attempting an action that was not allowed (such as trying to leave when it would require breaking a hold) or (iv) simply being in the game (to stop agents passing unnecessarily, and to encourage shorter transactions).

The interactions proceeds as follows: at each step an agent is chosen randomly, allowing them to choose an action, and both agents receive any immediate rewards; $R_{B}^{A}(s,d,s^{\prime })$ is the reward that agent A gets if agent B takes action *d* in state *s*, resulting in new state *s*′; we use subscripts to indicate the actor and superscripts to indicate the agent receiving the reward. The reason we choose agents randomly rather than enforcing alternation is to allow for the possibility of compound actions. Agents also have the option to pass. The interaction continues until one of the agents successfully exits. At this point, both agents receive a final reward, which is the value they assign to the objects that they hold.

The value function V(s)→R stores the expected return, under the assumption that the optimal action is taken at each iteration; where two or more actions had equal expected reward, one was chosen at random. To compute *V*, we maintain the expected return for (state, action) pairs, known as *Q* matrices $Q_{Y}^{X}\;:\;(S\times A)\to R$, where *X*, *Y* = A or B, and $Q_{X}^{Y}$ denotes the expected return for agent *Y* when agent *X* acts; there are thus four *Q* matrices, two for each agent. Both agents update their expectations at every iteration using Bellman’s equation [17,19]

2.1 $$Q_{X}^{Y}(s,d)=\sum_{s^{\prime }}P_{X}(s^{\prime }|s,d)(R_{X}^{Y}(s,d,s^{\prime })+\gamma V^{Y}(s^{\prime })).$$

where *P*_*X*_(*s*′| *s*, *d*) is the probability of moving to state *s*′ when action *d* is taken in state *s* by agent *X*, mostly *P*_*X*_ = 1, meaning that the intended action is always taken. If *P*_**exit**_ is set to be less than 1, then the effect is that the agent is not always successful in leaving at the end of a transaction. We call this a ‘slow exit’; it enables the other agent to react by attempting to take a hold if they wish to stop the first agent leaving. The parameter *γ* discounts future rewards, since the future is not fully predictable. The value *V* for agent A is (where *β* is the probability that agent A is chosen to carry out the next action, and thus allows us to vary the relative ‘reaction time’ of the agents)

2.2 $$V^{A}(s)=\beta \max_{d}Q_{A}^{A}(s,d)+(1-\beta )Q_{B}^{A}(s,e^{⋆}),$$

where

2.3 $$e_{s}^{⋆}=\underset{e}{\arg\max}Q_{B}^{B}(s,e)$$

For agent B, *β* is replaced by (1 − *β*). Thus we have two interleaved Markov decision processes seeking to optimize related competing functions, which converge to a shared solution.

Two extra parameters model the effect of errors and uncertainty:

**Action noise.** With a small probability the agent performs a different action (chosen uniformly at random from those allowable).
***Q* noise.** A small amount of zero mean Gaussian noise is added to the *Q* matrices at each iteration (this also deals with ties in choices should they occur).

Finally, we optionally include a slowing of the ‘exit’ action by reducing the probability *P*_*X*_ of the result of the exit action being an actual exit relative to taking a hold. In effect a low value means it takes several time steps to exit, and this gives the other agent time to react and stop the first agent from leaving, either by taking hold of an object held by that agent, or the agent themselves.

An implementation is available at https://github.com/smarsland/origin_money/tree/master/exchange.

## 3. Results

**Figure 3.**
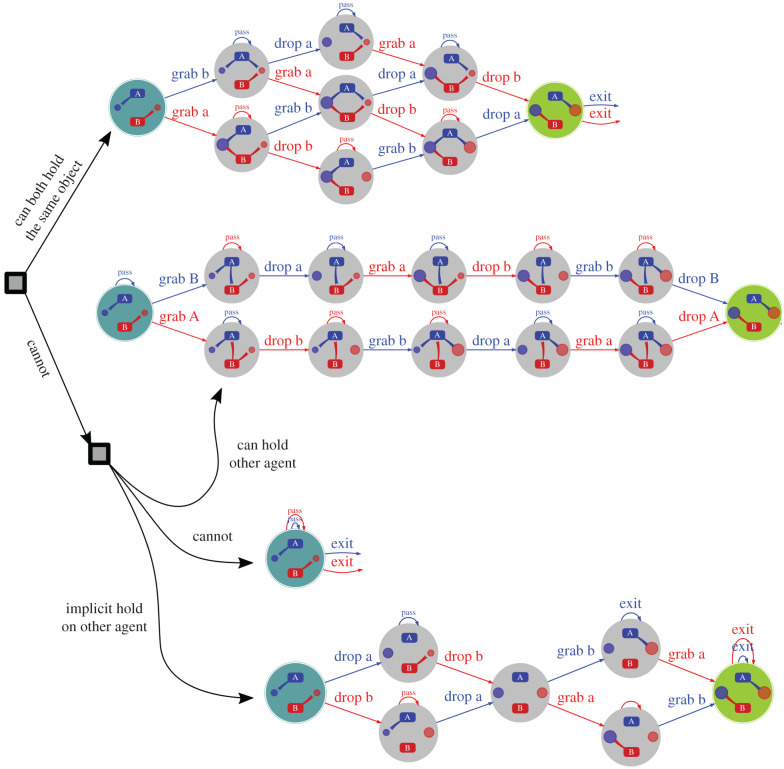
A tree showing some possible swap dances. Working from top to bottom: (i) there is a solution if both agents can hold an object simultaneously, or (ii) hold the other agent. However, there is not if (iii) the agents cannot do either of these. (iv) A fast, but unsafe, swap can be achieved if the agents believe that they can stop the other agent leaving (by taking hold of them) should they wish to. Dances are drawn from left to right. States are shown within grey circles, with the actions labelled on the arrows. These dances are all symmetric, depending on which agent starts, although the agent that moves at each step is chosen randomly. (Online version in colour.)

Our main aim was to see whether or not pairs of agents could find, and prefer, safe swapping interactions. We performed simulations using a computational model to investigate this, testing the effects of the various parameters. The *Q* and *V* matrices were initialized with zero mean Gaussian random numbers with standard deviation of the size of *Q* noise. One hundred runs of each set of model parameters were run for 1000 steps each, although the models converge far earlier, usually in less than 100 steps. In general, four different ‘dances’ (exchange interactions, i.e. optimal joint trajectories through state space) that result from different parameter choices were found. Figure 3 summarizes these dances. Each dance is optimal in the sense that it maximizes the total expected rewards of both parties. The dances are symmetric; in some sense, the only choice is who initiates the action. Three dances (and one failure) are shown, differing by which holds are allowed; we consider four different variants of the holds:

**All holds allowed.** This is the full space of 64 states.
**At most one agent holds an object.** If agents can hold an object at any given time (at most one of bits 1 and 4 and bits 3 and 6 can be set to 1), then there are only 36 allowable states.
**Agents cannot hold each other.** In this case, bits 2 and 5 are set to 0, and there are 2^4^ = 16 allowable states.
**At most one agent holds object** (and agents cannot hold each other). There are only 10 allowable states.

If the agents can both hold on to the same object, then it is possible for each to stop the other from leaving with both objects. The top line of the tree shows such a case. If the agents can only hold an object that the other is not holding, then they do not find a satisfactory way to swap, and simply leave with their original good. However, when agent holds are allowed, they can (second graph). One agent grabs the other, then drops its good, which the other collects before dropping its own, which is collected by the first agent, who then releases their hold.

Such holds enable safe exchange, and yet they are not typical of human interactions. However, they do not need to be explicit: the lowest branch in the tree shows that the opportunity for an agent to grab the other agent (or an object) if necessary, to stop them leaving, is sufficient to allow swaps again.^1^ Such swaps are slightly unsafe, but faster, and require less contact between agents.

The algorithm quickly and consistently converges to these same solutions over a large range of parameters (see the electronic supplementary material). We vary the perceived values of the goods, the penalties for failing to swap, or for taking too long to achieve an exchange, and how noise (error) affects the model. A few key points from our results are described below.

**The model is very stable.** Most dances appear consistently across a wide range of parameter values. This is particularly true of the solution where both agents hold the same object, which is by far the most common one found.
**Most solutions are symmetric.** Even among the extreme parameter value dances, most of them are entirely symmetric (such as those in figure 3). The agents take it in turns to perform an action and react as expected, which is why we call them dances. This remains the case even when the probability of one agent being selected to perform an action at each turn is biased (e.g. if $\beta \ll \frac{1}{2}$).
**Costs matter.** Including a cost to take hold of objects enables the ‘implicit’ (technically non-safe) solution to be found. Without a cost for failing, the agents will try to exit at any advantageous situation, and also to break holds. However, a cost for staying (which aimed to eliminate redundant interactions) did not make any difference. The actual value of the costs does not matter so much as whether or not they exist at all. This is even the case for the cost of failing.
**Perceived value does not matter until it is extreme.** We experimented with changing the value of the desired good (the one initially held by the other agent). Even when this value drops close to the value of good they are holding, the two agents swap, until the costs of the exchange drive the total payoff below the value of their current object, when the agents simply exit. For example, if the agents can hold both goods at once (multiholds), even when the higher-value good is worth 1.2, just 0.2 above the lower-value good, the two agents find a swap dance 100% of the time. For the risky implicit swap, the higher-value good needs to be worth double the lower-value one or the agents prefer to simply exit without exchanging.
**Asymmetric perceived values do not stop swaps.** If the perceived values change asymmetrically, so that one agent receives a lower reward than the other, and potentially even loses on the exchange, they can still be forced into the exchange if the other agent acts first and establishes a hold. As will be discussed shortly, this is particularly relevant to a model of shopping, where agents presumably place the same value on one good—money—while the second good is more highly valued by the buyer than the seller.
**Discounting is important.** Discounting means that future rewards are reduced in value. Stronger discounting, particularly for the agent-hold models, leads the agents to take risks that can leave them without any objects. In general, *γ* has to drop below 0.8 before the risks lead to agents not being prepared to perform the exchange, even allowing for the risk of the other agent defecting.
**Noise does not affect the model.** Even with no noise agents generally find an optimal solution, even though noise is what enables agents to take an action that is not the one they currently believe is optimal, and thus to search the space further. Further, optimal solutions remain the same despite large (25%) noise values for both *Q* and action noises.

**Figure 4.**
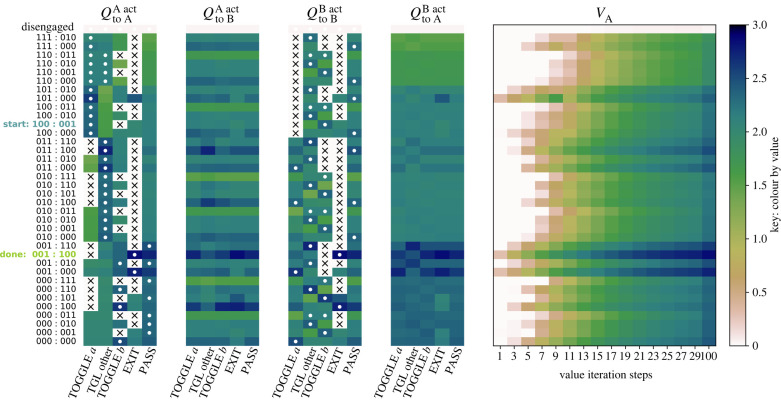
Values in the expected reward matrices for the two agents for the swap dance with an implicit hold. The colour map is shown on the right. Crosses indicate impossible actions (for example, an agent cannot exit when being held), and white circles denote the preferred action for an agent based on its own rewards. There are four *Q* matrices, which contain the learned values, showing what each agent expects based on the actions of itself and the other agent, for each of the five actions. The *V* matrix shows the learning process working; it records the start and every second iteration up to 30, and the 100th iteration, for agent A; although it is not enforced, these matrices are largely symmetric. Scores for the final *V*_A_ and $Q_{A}^{B}$ are given for the case when object a is worth 1 and object b worth 4 to agent A, and vice versa for agent B. (Online version in colour.)

Figure 4 shows the *Q* and *V* matrices identified by a pair of agents that learn to swap using an ‘implicit’ hold, i.e. the bottom row of figure 3. Other examples are given in the electronic supplementary material. The *Q* matrices show the learned values of the expected reward for each agent, based on which agent is acting, for each action in each state, with the optima for the agent’s own expected reward marked with a white circle. The *V* for agent A records the updating of the expected reward for each state for the first few iterations of learning, and after 100 steps. The trajectories that describe the full swap begin at the state denoted 100 : 001, in which A has object a and B has object b, and finish in 001 : 100. The entire search space and other examples are given in the electronic supplementary material.

## 4. Discussion

We have shown that two strangers can safely exchange objects without the involvement of a trusted third party. However, in order to establish a truly safe exchange a hold is the fundamental requirement. In our model, this can be of the other agent, or of one or both of the objects. In any of these cases, stable dances are found and the agents successfully swap safely across a wide range of parameter values in the model. However, such dances are seldom seen in the countless exchange interactions that take place every day across the world.

Since safe exchange is possible, it is natural to ask why it is not generally seen in practice: the act of exchange is almost always carried out with risk, and yet without conscious thought. The complex choreography required may well be part of the answer. The fast, unsafe swap at the bottom of figure 3 most closely matches the majority of human exchanges. We postulate that this threatened hold is the internalized state on which everyday interactions implicitly rely; they have lower cost in terms of speed and imposition on personal space, and they are sufficient the vast majority of the time. It is only when exchanges are outside common norms that further considerations are necessary: witness film and fiction portrayals of drug deals and kidnapping hand-offs, or more prosaically, the purchase of souvenirs in a foreign market, where the lack of familiarity and language exascerbates the feelings of risk.

However, this is not the whole story. In mercantile exchange, the arena of swapping (the shop or marketplace) makes the differing roles of the two agents clear, in that one has to enter the premises of the other in order to perform the exchange. Some shops go to great lengths to establish an implicit hold: in many supermarkets, one enters through a one-way turnstile, and can only leave via the checkouts. It seems likely that social pressure and physical proximity have the same effect in other environments, and in this way the implicit hold is ingrained.

Two modern variations of shopping differ markedly from this. One is standard online (and earlier, mail order) shopping, while the other is the use of auction sites. In these, the risk is usually placed on the purchaser, who pays in advance (enables the retailer to place a hold on the money) in order to make the purchase, and then waits for the transaction to clear, and the goods to be sent (the hold on the goods to be relinquished). Note that this is included in our computational model by allowing asymmetric values for the higher-rated good. For an established shop, people place implicit trust in the vendor through the reputation signalling of a high-quality website and the ability to take credit card payments, while the latter deal with the true anonymity of many of their traders through a reputation mechanism that is an implementation of indirect reciprocity: ‘The money that fuels the engines of indirect reciprocity is reputation’ [20]. The cost of such fuel is the need to trust the reputation of the seller, as maintained by the website: reputations require powerful third parties to enforce, and track scores.

When considering merchantile exchange, the other key component is that one of the goods that are swapped is money, which (despite being itself an inherently worthless token) has the same perceived value to both agents. Experiments with our computational model show that provided the other good has a difference in perceived value to the agent initiating the exchange process, this does not matter. The asymmetry in the desire for the exchange can be considered to be offset by the reduced risk on behalf of the seller, who controls the implicit hold.

We mentioned earlier that exchange can be seen as an extension of the reciprocity models of evolutionary game theory. The exchanges we have studied here are anonymous and one-shot. Rand & Nowak [11] suggest that cooperation in this setting might be a side-effect of the benefits of reciprocal cooperation [9,21], either biologically or culturally. On their account, one-shot cooperative systems can be gamed, and thieves can flourish temporarily, but the overall cost is lower than the disadvantage of slower, more intrusive, exchange protocols. However, our analysis shows that it is possible to achieve mutually acceptable exchanges without risk.

In common with the reciprocity literature, it is natural to ask whether this is a purely human phenomenon. The ‘dances’ that we have found are fairly simple, and are found consistently over large ranges of parameters. The agents greedily attempt to optimize their own payoff without strategizing, and consensus emerges from this. Note that while we have used value iteration in two competing Markov decision processes to study the space, this is not to suggest that the ability to apply Bellman’s equations is required in order to learn to exchange; any form of search over the space of actions would be sufficient, although it may take longer to reach a good solution.

The affordances required to implement swapping dances are simple: the ability to hold, and some very low-level reinforcement learning or the selection of appropriate behaviour via evolution; many animals could identify such dances. Recent work has shown that reciprocal trading—a far more complicated interaction—occurs in many animals, including rats [22] and chimpanzees [23] (see also discussions about egg trading in hermaphrodites [24], and descriptions of biological markets [25]). Despite this, exchange remains a human-only behaviour that we accomplish efficiently, yet not safely, without considering its risks. Whether this restriction to humans arises from a lack of incentive for animals to swap safely, an inability to perform multiple holds simultaneously, or some other issue that we have not considered, remains an open question.

## Data Availability

Data are available at https://github.com/smarsland/origin_money/tree/master/exchange. Electronic supplementary material is available online [26].

## Endnote

1 A variation on the shared holds uses locks: agent A applies a lock to object a, agent B does the same for object b. Agent A adds a lock to object b, and agent B reciprocates (this is analogous to them both holding both objects). Agent B can then unlock object b, and agent A can unlock object a, which facilitates the swap. Interestingly, in this variant, it is possible for one or both objects to end up unusable, which cannot happen in the holding version.
